# Role of Nuclear Receptors in Controlling Erythropoiesis

**DOI:** 10.3390/ijms23052800

**Published:** 2022-03-03

**Authors:** Valentina Pastori, Serena Pozzi, Agata Labedz, Sajeela Ahmed, Antonella Ellena Ronchi

**Affiliations:** Department of Biotechnology and Biosciences, University of Milano-Bicocca, 20126 Milan, Italy; valentina.pastori@unimib.it (V.P.); s.pozzi49@campus.unimib.it (S.P.); agata.labedz@unimib.it (A.L.); sajeela.ahmed@unimib.it (S.A.)

**Keywords:** nuclear receptors, erythropoiesis, stress erythropoiesis, globins genes expression during development

## Abstract

Nuclear receptors (NRs), are a wide family of ligand-regulated transcription factors sharing a common modular structure composed by an N-terminal domain and a ligand-binding domain connected by a short hinge linker to a DNA-binding domain. NRs are involved in many physiological processes, including metabolism, reproduction and development. Most of them respond to small lipophilic ligands, such as steroids, retinoids, and phospholipids, which act as conformational switches. Some NRs are still “orphan” and the search for their ligands is still ongoing. Upon DNA binding, NRs can act both as transcriptional activators or repressors of their target genes. Theoretically, the possibility to modulate NRs activity with small molecules makes them ideal therapeutic targets, although the complexity of their signaling makes drug design challenging. In this review, we discuss the role of NRs in erythropoiesis, in both homeostatic and stress conditions. This knowledge is important in view of modulating red blood cells production in disease conditions, such as anemias, and for the expansion of erythroid cells in culture for research purposes and for reaching the long-term goal of cultured blood for transfusion.

## 1. Introduction

### 1.1. Erythropoiesis

Erythropoiesis leads to the formation of the most abundant cells of our body, red blood cells (RBCs), specialized in the oxygen transport to tissues and in the removal of carbon dioxide. In steady state conditions, RBCs are produced at a rate of about 2 × 10^6^ per second. This number can greatly increase upon stress conditions. During development, their production becomes indispensable as soon as the growing size of the embryo makes passive gases exchange insufficient.

Different waves of erythropoiesis take place during development: the first two (hematopoietic stem cell (HSC)-independent) originate from the yolk sac (YS), whereas definitive, HSC-dependent erythropoiesis, is generated from HSCs emerging from the aorta-gonad-mesonephros (AGM) region, the placenta and possibly from other anatomical sites [1]. The first erythroid cells are called primitive erythroid cells (EryP) and are released into the bloodstream while they are still nucleated. The second wave derives from erythroid-myeloid progenitors (EMPs) of YS origin that colonize the fetal liver and generate the first enucleated RBCs. Finally, definitive adult erythrocytes derive from HSCs that first colonize the fetal liver and then the bone marrow, the anatomical site of adult hematopoiesis.

The differentiation of RBCs from HSCs entails several steps leading to the appearance of erythroid-committed progenitors (erythroid burst- and colony-forming units, BFU-E and CFU-E) which give rise to proerythroblasts that progressively differentiate and mature into RBCs, through morphologically well-defined stages. Erythropoiesis is regulated by external signals provided by the microenvironment and by intrinsic factors that coordinate the expression of the genetic program typical of the erythroid cell type, including developmental stage-specific globins.

### 1.2. Nuclear Receptors

**Figure 1:**
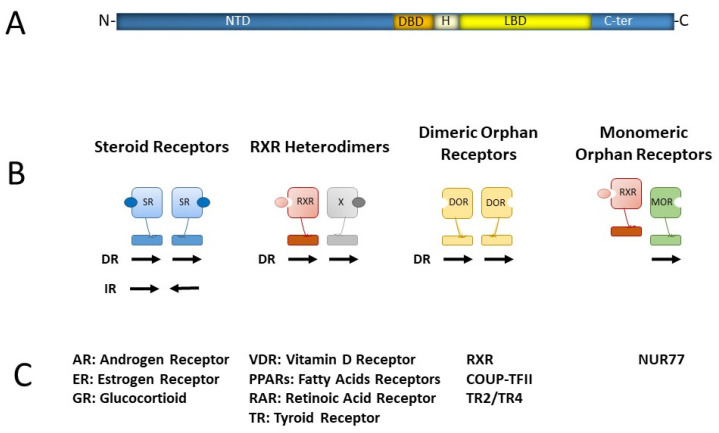
Nuclear receptors: (**A**) schematic structure of nuclear receptors. NTD: N-terminal domain; DBD: DNA-binding domain; H: hinge; LBD: ligand-binding domain. (**B**) The four classes of nuclear receptors. (**C**) The nuclear receptors acting at the different stages of erythropoiesis and discussed in the text are listed under the class they belong to.

The nuclear receptors, NRs, represent a family of evolutionary conserved proteins acting as ligand-activated transcription factors. NRs are classified according to their evolutionary distance in six subfamilies numbered from one to six and each divided in several groups indicated with letters [2]. They are composed by two main domains, a zinc-finger DNA-binding domain (DBD), necessary for the binding to their target regions on DNA (the response elements, RE), and a ligand-binding domain (LBD), Figure 1. Despite the presence of a structural LBD, for some NRs the ligand is still unknown, and they are then classified as “orphan receptors”. NRs present an extremely various pattern of expression and are translated into multiple isoforms with different transcriptional regulatory potential. Moreover, NRs can complex as homodimers or heterodimers and thus recruit different cofactors to promote the assembly of multiprotein complexes with different specificity and transcriptional output. An additional layer of plasticity is represented by their binding sites on DNA, which can be formed by monomeric sites or by two repeats separated by spacers of different length and with different possible orientations (direct repeats, DR, or inverted repeats, IR). Finally, NRs can bind to a wide spectrum of variant DNA consensus sequences. This flexibility generates a very complex combinatorial regulatory code [3,4,5] (Figure 1).

**Figure 2:**
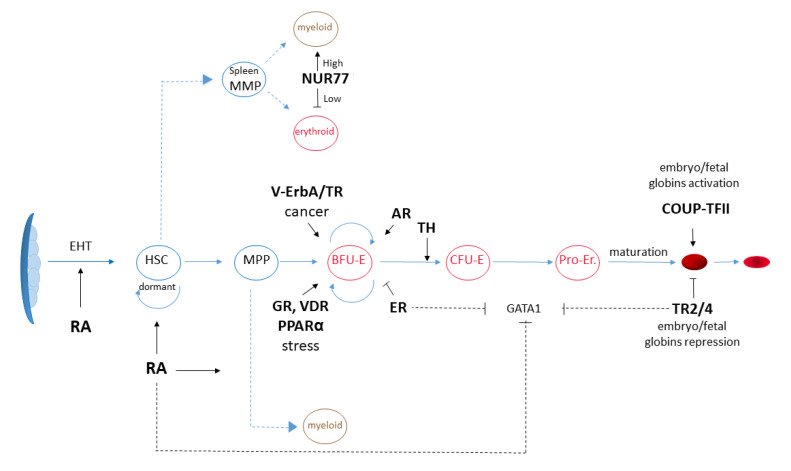
Schematic representation of the major NR signaling pathways affecting erythropoiesis and of their role in erythroid cell lineage specification and maturation. EHT: endothelial hematopoietic transition; HSC: hematopoietic stem cell; MMP: multiple myeloid progenitor; BFU-E: blast-forming unit, erythroid; CFU-E: colony-forming unit, erythroid; Pro Er: proerythroblast; MPPS: splenic multipotent progenitors; RA: retinoic acid; ER: estrogen receptor; AR: androgen receptor; GR: glucocorticoid receptor; GR: granulocytes; VDR: vitamin D receptor; PPARα: peroxisome proliferator-activated receptor; v-ErbA: mutated version of thyroid hormone receptor-α, responsible for avian erythroblastosis; T3: triiodothyronine; TR2/4: testicular receptors 2/4; COUP-TFII: chicken ovalbumin upstream promoter transcription factor II.

The aim of this review is to highlight the involvement of different nuclear receptor family members in different crucial steps for erythropoiesis, ideally following the trajectory leading from the emergency of hematopoietic stem cells to differentiated red blood cells (Figure 2). In particular, we will first focus on NRs having a role in the emergence of erythropoietin-dependent erythropoiesis in early development and on NRs involved in the control of the proliferation-differentiation balance of erythroid progenitors in steady state and stress conditions. Finally, we will introduce NRs important for the regulation of the differential expression of globins genes during development.

## 2. NRs Controlling Normal Erythropoiesis

### 2.1. The Developmental Control by Retinoic Acid: From the Emergence of HSCs to the Establishment of Erythropoietin-Dependent Erythropoiesis

Vitamin A acting through its main active metabolite, retinoic acid (RA), is essential for patterning during embryonic development and for adult tissue homeostasis [5]. RA signaling acts through two families of nuclear receptors: retinoic acid receptors (RARs) and retinoic X receptors (RXRs), including three members α, β and γ for each family. RXR and RAR assemble in RAR/RXR heterodimers, although RXR can also form homodimers or heterodimers with other nuclear receptors, such as the vitamin D receptor (VDR) or Peroxisome proliferator-activated receptors (PPARs). RAR/RXR recognize on DNA a specific consensus sequences named “RARE”, retinoic acid response element, composed by DRs with variable spacing (DR1, DR2, DR5) [6]. When they are bound to DNA, RARs act as a transcriptional repressor or activators by recruiting cofactors, thus allowing repression or activation of target genes [7,8]. In hematopoiesis, RA signaling is essential for the specification of embryonic hematopoietic stem cells (HSCs) from the haemogenic endothelium within the AGM region [9,10,11,12,13]. RA acquires a transient specific role in erythropoiesis in early fetal liver (E9.5–E10.5 in mouse), where it activates the expression of the Epo gene, coding for erythropoietin, the major hormone stimulating erythroid cells production, thus marking the switch from yolk-sac-derived, Epo-independent, to the HSC-derived, Epo-dependent erythropoiesis, see below [14]. In adults, RA is important for maintaining HSCs in a dormant state (dHSCs), preventing their activation and entry into the cell cycle in response to activating signals [15].

The correlation between Vitamin A deficiency and anemia is well known in humans and in different animal models. However, the molecular mechanisms of RA action is not clearly understood and it likely involves both direct and indirect effects on erythroid progenitors, mainly related to the modulation of iron metabolism and inflammation [16].

Early studies on ex vivo human cultures showed that RA stimulates granulocytic differentiation at the expenses of the erythroid differentiation program by inhibiting the expression of GATA1, the master erythroid transcription factor, in early erythroid progenitors [17]. In line with this, defective RA/RARα axis downstream to the PML-RARα translocation causes acute promyelocytic leukemia due to a failure of promyelocytes differentiation. The pharmacological administration of RA together with arsenic trioxide, restores terminal differentiation of these leukemic progenitors and it is considered the most successful example of “differentiation therapy” [18].

#### Mouse Models

The role of RAR/RXR has been addressed in detail by several mouse models including loss of function mutants, mutants in specific protein domains and compound double or triple mutants. RA deficiency caused by the knock-out of the RALDH2 gene (retinaldehyde dehydrogenase 2), which catalyzes the synthesis of retinoic acid (RA) from retinaldehyde, leads to death at E10.5 because of multiple defects, including yolk sac malformations. Its conditional deletion in the endothelium (VE-CadherinCre/RALDH2flox model) hampers the formation of HSCs from the hemogenic endothelium [10]. Surprisingly, the RARα knock-out itself leads to a normal development, possibly because of compensatory effects by other RARs [19]. Instead, RARγ knock-out mice are anemic and prone to develop myeloplastic syndromes, although the RARγ conditional erythroid-specific deletion (EpoRCre/RARγ−/−flox model) has no effect on erythropoiesis, suggesting a non-cell-autonomous defect of erythroid cells [20,21]. RXRα deficient mice exhibit a transient anemia (E9.5–E11.5 in mouse) due to the inability to induce erythropoietin (Epo) gene expression, but they look normal at E12.5. The transient nature of anemia likely relies on the differential contribution during development of two regulatory regions controlling the expression of the Epo gene: the enhancer containing an RXR/RAR binding site (the DR2 site) adjacent to a hypoxia response element bound by the hypoxia-inducible factor (HIF1) and the promoter, containing a GATA binding site. In the hypoxic early fetal liver environment, the enhancer plays a dominant role in Epo gene transcription. However, this same element becomes dispensable at later stages, when Epo gene expression mainly depends on the GATA site within the promoter [14,22].

The RXRα/RXRβ double mutant embryos die between E9.5 and E10.5 due to placental abnormalities and multiple malformations [23]. Finally, the double RXRα/RXRγ and RXRβ/RXRγ compound knock-out have a phenotype identical to that of the single RXRα and RXRγ knock-out, respectively. Surprisingly, the triple RXRα+/−/RXRβ−/−/RXRγ−/− mutant is vital, suggesting a large functional redundancy of RXRs [24].

### 2.2. NRs Affecting Erythroid Differentiation

#### 2.2.1. The Thyroid Hormone Receptor: NR1A1/2

Triiodothyronine (T3), the active form of the thyroid hormone, TH, exerts its action by binding to the nuclear thyroid hormone receptors (TRs), encoded by the TRα and TRβ loci, which generate different isoforms with different pattern of expression and functions [25,26,27].

The role of TH and TRs in erythropoiesis has been suggested during years by the observation that T3 stimulates erythropoiesis in humans and in animal models [28,29]. Moreover, the avian erythroblastosis v-ErbA oncoprotein, a dominant negative form of c-ErbA/TRα, promotes erythroid progenitors’ expansion while blocking terminal differentiation in chicken cells. Under physiological conditions, c-ErbA/TRα acts as a switch: in the absence of a ligand, it supports the proliferation of erythroid progenitors by cooperating with the stem cell factor receptor c-kit, whereas in response to the T3 ligand it unlocks synchronous erythroid terminal differentiation [30,31,32]. Because of TRs’ role in erythropoiesis, in patients, hypothyroidism is frequently associated with mild anemia [31,32,33] and erythroid cultures from hypothyroid patient cells show delayed differentiation of progenitors, high levels of c-kit (expressed on the surface of immature cells) and reduced GpA (glycophorin A, expressed by more mature erythroid cells) [34]. On the contrary, hyperthyroidism is associated with erythrocytosis [35].

##### Mouse Models

TRα1 knock-out mice show defective fetal and adult erythropoiesis, with a decreased number of early erythroid progenitors in the fetal liver and an impaired stress erythropoiesis response in the adults [36]. This phenotype is similar to that of the mouse model (PV/+ mice [37]), carrying a copy of the dominant negative frame-shift mutation, originally identified in patients, which abolishes the triiodothyronine (T3)-binding domain. However, the underlying mechanism is probably different in the two cases: the lack of TRα1 would lead to the loss of the regulation of erythroid TRα1 target genes, whereas the dominant TRα1PV protein main action consists in the repression of Gata1 expression [37]. Interestingly, the cross of PV/+ mice with mice expressing a mutant version of the corepressor NCOR1, unable to bind to the dominant negative PV protein, partially rescues terminal erythroid differentiation, further indicating that the action of the TRα1PV protein involves transcriptional repression [38].

As for TRβ, GWAS studies identified variants [39] and mutations [40] in the TRβ locus associated to red blood cells parameters, although anemia was not originally observed in patients with TRβ mutations [41]. The role of TRβ and of its coactivator NCOA4 in stimulating terminal differentiation has been shown in primary cells and in anemia mouse models, following the administration of TRβ agonists [42].

Interestingly, the NCOA4 knock-out results in transient anemia during embryonic development but this phenotype must take into account the role of NCOA4 in regulating iron metabolism that could, in turn, contribute to regulate red cells production [42,43]. The discrepancy of the results obtained in constitutive versus tamoxifen-induced NCOA4 knock-out mice add a further layer of complexity in the interpretation of the role of NCOA4 in erythropoiesis [44,45].

The mouse TRβ knock-out model has a normal erythropoiesis [46,47,48,49], whereas TRα1 knock-out in mice results in a reduced number of erythroid progenitors in a cell-autonomous way [37]. Interestingly, TH and TRα, but not TRβ, are specifically required for normal spleen erythropoiesis during early postnatal development [50], suggesting a predominant TRα role in early development. Finally, although a role of TH in stimulating hemoglobin production it has long been known [29], a recent observation describes the specific effect of TRIAC, a bioactive thyroid hormone metabolite, in the direct regulation of the embryonic ζ-globin gene expression [51].

### 2.3. The Complex Role of Sexual Hormones in Erythropoiesis

Several reports have demonstrated over the years that the administration of sex hormones (estrogens, progesterone and testosterone) have an impact on erythropoiesis (see below). The molecular basis of their action, however, is not completely clear and very likely relies, at the physiological level, on both cell-autonomous and non-cell-autonomous mechanisms. The very general message from the available data is that whereas testosterone stimulates RBCs production, estrogen has the opposite effect.

#### 2.3.1. Estrogen Receptors (ERα: NR3A1 and ERβ: NR3A2)

Estradiol promotes human stem/progenitor cells expansion [52] and estrogen receptor alpha (ERα) is clearly required for hematopoietic differentiation of human stem cells [53]. The first experiments based on the treatment of human and animal erythroid cultures with estrogen instead suggested that this female hormone inhibits erythropoiesis [54,55]. These experiments were confirmed in vivo, where high dosage of estrogens causes anemia in humans and animal models [54,56]. Interestingly, it has been observed that in people living at high altitudes, females are more protected than males against chronic mountain sickness erythrocytosis (CMS or Monge’s disease); of note, the incidence of CMS clearly increases after menopause, confirming the link with estrogen levels [57]. The same study reported that ER acts by repressing GATA1, with subsequent erythroid progenitor apoptosis, as originally observed by G. Blobel and colleagues [58,59]. These authors demonstrated that GATA1 repression by ER occurs in a ligand-dependent manner by direct protein–protein interaction.

##### Mouse Models

Mouse knock-out models carrying loss of function mutations of ERα, ERβ or both show, as expected, severe reproductive phenotypes, with other systems being also affected, such as bone, brain, immune, adipose and cardiovascular, but no overt erythroid defects have been described so far [60,61,62].

Regarding the hematopoietic system, ERβ knock-out mice develop a chronic myeloproliferative disease during aging, similar to human chronic myeloid leukemia, uncovering the ERβ involvement in the differentiation of pluripotent hematopoietic progenitor cells [63].

#### 2.3.2. Androgen Receptor (AR: NR3C)

The first evidence of a role of AR in erythropoiesis derives from the observation of the sexual difference in hematocrit [64]. The addition of testosterone to ex vivo erythroid progenitors confirmed that this hormone supports their expansion [65] and the in vivo administration of testosterone to different animal models supported this first finding leading to the development of androgen-based therapies to treat anemia [66]. The rationale of these approaches is that AR induces serum Epo [67,68]. Recent bone marrow reconstitution experiments with cells from mice lacking the DNA-binding domain of AR (ARΔZF2) suggested that the DNA-binding domain is indeed required to stimulate kidney erythropoietin [69].

#### 2.3.3. Progesterone Receptor (PR: NR3C3)

Progesterone was originally reported to increase fetal hemoglobin (HbF) in fetal calf liver erythroid cells and adult human erythroid progenitors, when administrated together with Epo [70,71]. In line with these results, uteroferrin (UF), a progesterone-induced acid phosphatase was shown to enhance fetal erythropoiesis [72].

## 3. NRs Controlling Stress Erythropoiesis Response

In steady state condition, about 10^11^ RBCs per day are produced to maintain homeostasis but this number can increase dramatically in response to hypoxia. This phenomenon is known as “stress erythropoiesis” [73,74].

Two NRs play a fundamental role in stress erythropoiesis: the glucocorticoid receptor (GR, NR3C1) and the Vitamin D receptor (VDR: NR1I1).

### 3.1. The Glucocorticoid Receptor (GR: NR3C1)

The glucocorticoid receptor (GR) is the constitutively expressed receptor of cortisol and other glucocorticoids (GC). Upon ligand binding, GR homodimerizes and is transferred to the nucleus, where it activates its target genes. In the absence of ligand, GR can bind to other transcription factors, such as AP1 and NFkB, or signal transducers, such as TGFβ and STATs, activating or inhibiting them [75,76,77]. Because of the combinatorial assembly of different GR complexes, depending on cellular and physiological contexts, GR controls several gene networks [78,79,80,81]. Cortisol and synthetic glucocorticoids, such as dexamethasone (DEX), are known regulators of erythroid progenitors, in vitro and in vivo [82,83,84]: DEX is currently used together with Epo and SCF to sustain the expansion of erythroid progenitors and to delay their terminal differentiation in liquid cultures, a condition mimicking stress erythropoiesis [84,85,86]. Moreover, pathological conditions altering glucocoticoids metabolism are associated with dysregulated erythropoiesis: insufficient adrenal corticosteroid production is often associated with anemia (Morbus Addison), whereas patients with elevated GC levels (Cushing’s syndrome) have increased red blood cells count, hemoglobin, and hematocrit [87]. In the clinical practice, DEX has been used to treat Diamond–Blackfan anemia (DBA). Recent work based on single cell analysis of bone marrow progenitors from DBA patients suggests that DBA cells fail to activate the endogenous glucocorticoid-pathway of stress erythropoiesis, which sustains the expansion of erythroid progenitors [88]. These authors suggest that the administration of DEX to DBA cells help restoring a proper stress response rather than re-establishing a normal erythropoiesis. This would explain the therapeutic effect of DEX on DBA patients, although with heterogeneous results [89,90]. In this regard, the search for GR single nucleotide polymorphisms (SNPs) associated with DBA response to glucocorticoids failed so far to find significant associations although it is possible that some SNPs could influence the onset of the disease [91].

#### Mouse Models

GR knock-out mice die perinatally, preventing the analysis of adult postnatal erythropoiesis [92]. However, adult erythroid cells from knock-out E14.5 fetal livers fail to expand in ex vivo cultures and undergo accelerated differentiation [93]. To assess GR-deficient erythropoiesis in vivo, mice carrying a GR mutation in the homodimerization domain, unable to bind DNA, were generated [94]. These animals grow to adulthood and are not anemic but fail to recover from induced anemias, a response sustained by stress erythropoiesis [93]. GR synergizes with another nuclear hormone receptor, PPARα, to enhance BFU-E production: both in vitro and in vivo, PPARα agonists and glucocorticoids enhance RBCs production [95]. Interestingly, PPARα knock-out mice have no defects in steady state or stress erythropoiesis. However, PPARα wild type but not PPARα−/− respond to PPARα agonists under induced acute haemolytic anemia. The observation that PPARα is recruited to GR-adjacent sites on chromatin suggests that PPARα could facilitate GR-dependent BFU-E proliferation [95], thus explaining the above result.

### 3.2. Vitamin D Receptor (VDR: NR1I1)

The vitamin receptor, VDR, is activated upon binding of Vitamin D3, which induces its translocation into the nucleus and its heterodimerization with RXR. The VDR/RXR complex recruits the transcriptional coregulatory factors that activate the vitamin D hormone-regulated genes [96].

Several observations associate vitamin D deficiency with anemia and vitamin D3 is known to improve anemia condition [97,98]. Vitamin D supports erythropoiesis by increasing burst-forming unit-erythroid (BFU-E) proliferation and has a synergistic effect with Epo and GR to further enhance erythroid progenitor cell proliferation [99,100,101]. Importantly, VDR is expressed during fetal and adult, but not embryonic erythropoiesis in early cKit^+^CD71^low/neg^ erythroid definitive progenitors and it is progressively downregulated during erythroid maturation [100]. According to this expression pattern, the vitamin D3 agonist calcitriol increases the proliferation of these progenitors and delays their terminal maturation. Although the targets of VDR in erythroid cells are still unknown, by analogy with other cell types where Vitamin D controls cell differentiation, it is plausible that genes essential to cell cycle progression are involved in erythroid progenitors’ expansion [102,103].

#### Mouse Models

In mice, the lack of VDR results in a perturbed HSCs homeostasis and in an increased propensity to develop leukemia caused by a block in myeloid differentiation [104], with no overt effects on steady-state erythropoiesis [105,106,107]. The synergistic effect of GR and VRD in supporting erythroid progenitors [100] raises the hypothesis that VDR could be involved in the stress erythropoiesis response. Indeed, a direct regulation of glucocorticoids on the transcriptional activation of VDR has been described in systems other than erythroid cells [108,109,110].

## 4. Orphan Nuclear Receptors

Orphan nuclear receptors owe their name to the absence of a known ligand. The resolution of their crystallographic structure revealed that in the majority of cases they possess an auto-inhibitory structure with side chains occupying the ligand pocket, thus preventing their activation by ligands. In some cases, as discussed below, they can nevertheless be activated by supramolecular doses of retinoic acid or by small molecules.

### 4.1. The Orphan Nuclear Receptors Controlling the Hemoglobin Switching

During development, the different waves of erythropoiesis generate RBCs characterized by the expression of different globin chains producing different hemoglobin tetramers. This phenomenon, known as hemoglobin switching, has an important clinical relevance since it is known that the reactivation of the fetal globin gene γ in adult cells could substitute for the defective adult globin gene β, whose mutations are the cause of β-hemoglobinopathies, the most common monogenic disease [111].

#### 4.1.1. Testicular Receptors 2 and 4 (TR2: NR2C1; TR4: NR2C2)

TR2 and TR4 are expressed in many tissues, including erythroid cells. They can form homodimers or heterodimers with one another and recognize on DNA directly repeated (DR) AGGTCA sequences, separated by 0–6 nucleotides. The involvement of the testicular receptor 2/4 (TR2/TR4) heterodimer in the regulation of the transition from embryo/fetal to adult globins expression was first described when these two proteins were identified as part of the DRED (direct repeat erythroid-definitive) complex, binding to and repressing the embryonic ε and fetal γ-globin promoters in adult cells [112,113]. Within the large macromolecular repressor complex DRED, TR2 and TR4 act as the core DNA-binding subunits. In addition to binding to the β-locus, TR2 and TR4 also bind to an evolutionally conserved DR element within the GATA1 hematopoietic enhancer (G1HE) and directly repress GATA1 transcription, suggesting that GATA1 may be directly silenced by TR2/TR4 during terminal erythroid maturation [114].

##### Mouse Models

The direct regulation of GATA1 expression by TR2 and TR4 described above, would explain why transgenic mice overexpressing TR4 alone (TgTR4) or both TR4 and TR2 (TgTR2/TR4) present a transient midembryonic anemia and defects in primitive erythroid precursor formation, which could not be explained simply by the effects of the TR2/TR4 protein complex on globins gene transcription [114].

Mice lacking TR2 are viable and show no evident mutant phenotypes, while TR4 germline mutants display reproductive and neurological deficiencies [115]. However, the careful analysis of erythropoiesis in heterozygous TR4+/− mutant erythroid cells evidenced reduced proliferation and incomplete differentiation [116]. Due to the early embryonic lethality of the double knock-out mice [117], erythropoiesis was studied in a conditional knock-out model in bone marrow cells induced to erythroid differentiation [115]. In these cells, the double TR2/TR4 ablation induced an embryonic globins increase accompanied by an impaired erythroid differentiation, confirming that beside their role in the hemoglobin switching, TR2 and TR4 have a broader role in erythroid differentiation.

#### 4.1.2. Chicken Ovalbumin Upstream Promoter Transcription Factor II (COUP-TFII, NR2F2)

NR2F2 owes its more common alias, COUP-TFII (chicken ovalbumin upstream promoter transcriptional factor II) to the name of the promoter where it was first identified to bind as transcription factor [118]. Structural data suggest that the COUP-TFII protein is capable of an autorepressive conformation, due to side chain amino acids occupying its own ligand pocket [119]. Nevertheless, it can be activated by supraphysiological concentration of retinoids and synthetic agonists and antagonist of COUP-TFII have been recently published [120,121,122]. COUP-TFII is essential for many biological processes, as demonstrated by the early embryonic lethality (around E9.5–E10) of COUP-TFII knock-out mice [118]. Conditional knock-out models further demonstrated the requirement of COUP-TFII in different developmental programs, including angiogenesis and cardiac cells specification [123]. Within the erythroid lineage, COUP-TFII is expressed in the early embryo in the yolk sac and in fetal liver and it declines around day E12.5 [124,125]. Similarly to TR2 and TR4, its involvement in the control of the hemoglobin switching, was first discovered because of its ability to bind to the DR1 DNA sequence within the γ-globin promoter [125,126,127]. What is more, COUP-TFII overexpression in adult cells specifically activates γ-globin, overcoming the repressive adult cellular environment [124]. Chromatin immunoprecipitation experiments confirmed the ability of COUP-TFII to bind to various positions within the locus control region (LCR) of the β-locus, with a pattern overlapping to that of Bcl11a-XL, a known repressor of γ-globin expression [128,129]. This last evidence suggests that the same DNA binding sites could be bound by a repressor or an activator at different times during development.

### 4.2. Nurr77 (NR4A1) and the Negative Control of the Erythroid Potential of Murine Splenic Progenitors

The NR subfamily 4 (NR4A) group contains three proteins with no known physiologic ligands: NR4A1 (Nur77/Tr3), NR4A2 (Nurr1), and NR4A3 (Nor1) [130,131,132,133]. Their crystallographic structure suggests that the ligand binding pocket is occupied by hydrophobic amino acid side chains that preclude the binding with endogenous ligands [134]. NR4s can bind DNA as monomers, homodimers, heterodimers with RXR (NR4A1 and NR4A2) or in complex with non-NR proteins. This flexibility allows NR4A proteins to regulate the expression of diverse targets in different cell types and accounts for their complex role in tumorigenesis, where they can act as tumor suppressors or oncogenes depending on the context [131,132]. In hematopoiesis, NR4As regulate the differentiation and function of various lymphoid [135,136] and myeloid cells [137,138].

#### Mouse Models

The involvement of NR4As in the repression of mouse spleen erythropoiesis has been highlighted by the observation that the NR4A1 knock-out mice have higher numbers of erythroid progenitors in the spleen. Indeed, NR4A1 regulates the potential of splenic multipotent progenitors (MPPS: cKit^+^CD71^low^CD24^high^ cells): NR4A1 expressing cells will differentiate into myeloid lineages, whereas NR4A1 nonexpressing cells will become erythroid cells [139]. Interestingly, in NR4A1 knock-out mice the number of erythroid progenitors in the bone marrow is normal. This “niche-specific” effect likely relies on the different expression level of NR4A1 in the two compartments: the NR4A1 level is higher in spleen than in bone marrow and this different requirement would render these cells more sensitive to its depletion [139]. The abnormal expansion of the myeloid compartment in the double NR4A1/NR4A3 knock-out mice results in the development of acute myeloid leukemia within the first months of life [137].

## 5. Conclusions

Various members of the NRs family play a role in erythropoiesis during development (as in the case of retinoic acid), in adult steady state (thyroid hormones receptors, sexual hormones) or under stress conditions (glucocorticoid receptors and vitamin D receptor) (Figure 2). In many cases, the treatment of human and animal erythroid cell cultures with NRs agonists (retinoic acid, dexamethasone, thyroid hormone, sexual hormones) has been the first evidence of the role of NRs signaling in different aspects of erythropoiesis. These in vitro data have been further supported by the observation that hormone/vitamin unbalance can cause anemia or erythrocytosis in patients and by the analysis of mutant mouse phenotypes. The overall emerging picture shows an extreme complexity of NRs signaling. This is due to NRs’ ability to both activate or repress transcription depending on the presence/absence of their ligand, on the cellular context, on the cellular niche and on the plethora of interacting proteins/signaling pathways. Despite this complexity, some agonists acting on NRs are already in use: the synthetic glucocorticoid dexamethasone and thyroid hormone are currently used to expand erythroid cultures, a precondition to reach the final goal to obtain large numbers of cultured red blood cells for transfusion [140]. Dexamethasone is also used in clinics to expand erythroid progenitors in DBA patients, although with heterogeneous results.

The advent of biochemical, biophysical, structural, and genomic approaches will help to better understand the role of NRs in physiological erythropoiesis and in disease conditions. This knowledge is essential to design therapeutic strategies based on NRs manipulation in order to improve the expansion/differentiation of erythroid cells.

## Data Availability

No new data were created or analyzed in this study. Data sharing is not applicable to this article.
